# Explainable machine learning for age-specific talent identification in Chinese youth football

**DOI:** 10.3389/fspor.2026.1912796

**Published:** 2026-09-02

**Authors:** Yan Tong, Jingyi Bi, Shixin Li, Luohang Chen, Chun Liu, Hengxin Chen

**Affiliations:** 1 School of Physical Education, Yunnan University, Kunming, China; 2 School of Life Sciences, Yunnan University, Kunming, China; 3 Kunming Institute of Zoology, University of Chinese Academy of Sciences, Beijing, China

**Keywords:** Chinese athletes, explainable machine learning, predictive modeling, talent identification, youth football

## Abstract

**Background and Aims:**

To identify elite youth football players in China, we developed an interpretable machine learning model.

**Methods:**

We modeled each age group separately to track changes in predictive factors across different age groups. A cross-sectional analysis included 398 players from the 2024 Yunnan Football Association Elite Training Camp (U8 to U12). All participants underwent a multidimensional assessment covering anthropometric, physical, technical, psychological, and match performance dimensions. In our prediction model, coaches' selection of provincial elite players was used as the outcome variable. Three classifiers: XGBoost, Random Forest, and Logistic Regression, were trained with hyperparameter tuning and 5-fold cross-validation. Then we used SHapley Additive exPlanations (SHAP) to interpret our models.

**Results:**

The best-performing model demonstrated high discriminative ability (test set ROC-AUC: 0.900–0.957). SHAP analysis indicated that dribbling speed and standing long jump consistently mattered at all ages, while sprint performance were especially important for the U8 and U10 groups. Notably, for U12 males, left-foot shooting skill was the strongest predictor, while for U12 females, tactical awareness during matches emerged as most important. Psychological traits contributed little predictive value. When we applied retrospectively to national selection data, the U12M model showed fair agreement (*K* = 0.269) but identified 40%–47% of national elites and highlighted several overlooked high-potential players.

## Introduction

Talent Identification (TID) in youth football remains a global challenge. Its core objective is to accurately predict future elite performance to optimize the allocation of various resources for developing young players (1). Traditional methods primarily rely on coaches' subjective assessments, which are typically based on anthropometric data, physical fitness tests, or observations during matches (2). While this experience is invaluable, it is susceptible to coaches’ cognitive biases and may overemphasize short-term performance over a player's long-term potential (3, 4). As a result, many talented athletes risk being overlooked, while others with limited developmental ceilings may be promoted prematurely—a phenomenon often referred to as the “selection error problem” (5). Consequently, sports science is increasingly advocating for objective, multi-dimensional selection frameworks. Reilly, Williams, and their colleagues pioneered the development of a foundational model that elegantly and systematically addresses the interplay of physiological, psychological, sociological, technical, and tactical factors in talent development (1), but it is also incontrovertible that its application in talent selection remains fragmented (3). Moreover, the youth football domain produces vast amounts of high-dimensional data, which in itself presents a major analytical challenge. Hence, traditional methods such as Logistic Regression (LR) are ill-suited for capturing the complex, non-linear relationships between the many variables that determine elite potential (6).

To address the aforementioned challenges, the emergence of machine learning (ML) algorithms such as Random Forest (RF) and Extreme Gradient Boosting (XGBoost) has brought new opportunities. These methods excel at building nonlinear relationships in high-dimensional data without requiring strict distributional assumptions (7). Meanwhile, the advent of Explainable Artificial Intelligence (XAI) frameworks has helped us gain clearer insights into these models (8). Particularly, SHapley Additive ex Planations (SHAP) can assist coaches in better understanding and applying these models. SHAP can reveal the factors that have the greatest impact on overall predictions and also provide local explanations for individual players (9). In recent years, research in Europe has successfully applied these methods to football and handball, achieving higher predictive accuracy than traditional methods in identifying athletic talent (6).

While ML and XAI hold great promise, a relatively underexplored area is their development in dynamic contexts. Youth sports participation is typically divided by age, from childhood to early adolescence, during which athletes undergo significant changes in physical, cognitive, and technical factors (10). Predictors that are important at age 8, such as speed, may lose relative importance by age 12, being replaced by technical or tactical understanding (11). The differences between age groups indicate that the predictive features of talent are developmentally stage-related and not static. Therefore, theoretically, a “one-size-fits-all” prediction model does not align with athlete development. Research must not only build accurate models but also clarify how the importance of features varies across different age stages (12). Furthermore, the effectiveness of such models must be validated in practical selection processes.

Chinese football is developing rapidly in line with national strategy, providing the relevant context. Although a wealth of data on youth training and selection has been generated (13) the analysis methods for this data remain at a descriptive level, limiting predictive insights. Furthermore, influenced by specific sports cultures and educational systems, the pathways of youth selection and athlete development in China may differ significantly from those in the West. Therefore, applying explainable machine learning to Chinese football youth data can achieve two objectives: advancing methodological progress in talent identification; and providing concrete insights for policies and practices in China's burgeoning football environment (6, 9).

To address the aforementioned issues, we set four specific research objectives: (1) To identify the most important predictors of elite status across different age groups (U8, U10, U12M, U12F) using explainable ML. (2) To analyze how these features shift from childhood to early adolescence. (3) To validate the model against external higher-level selection outcomes (national team selection) and examine the consistency and differences between provincial and national selection criteria. (4) To identify players who might be overlooked in traditional selection processes (6). Through a complete workflow: data preprocessing, exploratory analysis, model training and interpretation using SHAP, and finally external validation. Our study provides a replicable framework for the application of XAI in youth sports and offers new insights into the development of football among Chinese adolescents of different age groups.

## Materials and methods

### Study design

We conducted a cross-sectional study, applying machine learning retrospectively to analyze data from talented youth soccer players in Yunnan Province, China. We divided players into four groups based on their age and gender: U8 (7.0-8.9 years), U10 (9.0-10.9 years), U12M (11.0-12.9 years), and U12F (11.0-12.9 years). Of note, precise birth dates were not available in the dataset, preventing the calculation of relative age quartiles or adjustment for Relative Age Effects (RAE) in the analysis. Based on the selection results of provincial expert coaches, players within each group were further divided into two competitive levels (elite vs. non-elite). After grouping, we implemented a multidimensional assessment for all players. The assessment as follows. Anthropometric indicators: height (HT, cm); body mass (BM, kg); body mass index (BMI, kg/m^2^). Basic physical ability tests (standing long jump, SLJ, m), and football skill tests: passing accuracy (PAC, score) and dribbling speed (DRI, s). Specific tests are implemented for different age groups: the U8 and U10 groups underwent a 10-meter sprint (SP10, s) to assess sprint performance, while the U12 groups underwent a 30-meter sprint (SP30, s). Additionally, shooting accuracy was assessed using left foot (LFS, score) and right foot (RFS, score) only for the U12 groups. Psychological profiling was conducted using the junior version of the Eysenck Personality Questionnaire (EPQ), from which scores for Extraversion (EXT) and Neuroticism (NEU) were derived. Finally, expert coaches provided subjective ratings on four match performance dimensions after observing small-sided games: technical (MT), physical (MP), tactical (MTA), and social (MSO) performance, each on a defined scale. The primary dependent variable was provincial elite selection status (yes/no), serving as the target for the classification machine learning and interpretability analyses. For the U12M group, a secondary dependent variable, national elite selection status (yes/no), was used for the subsequent validation analysis.

### Subjects

**Figure 1:**
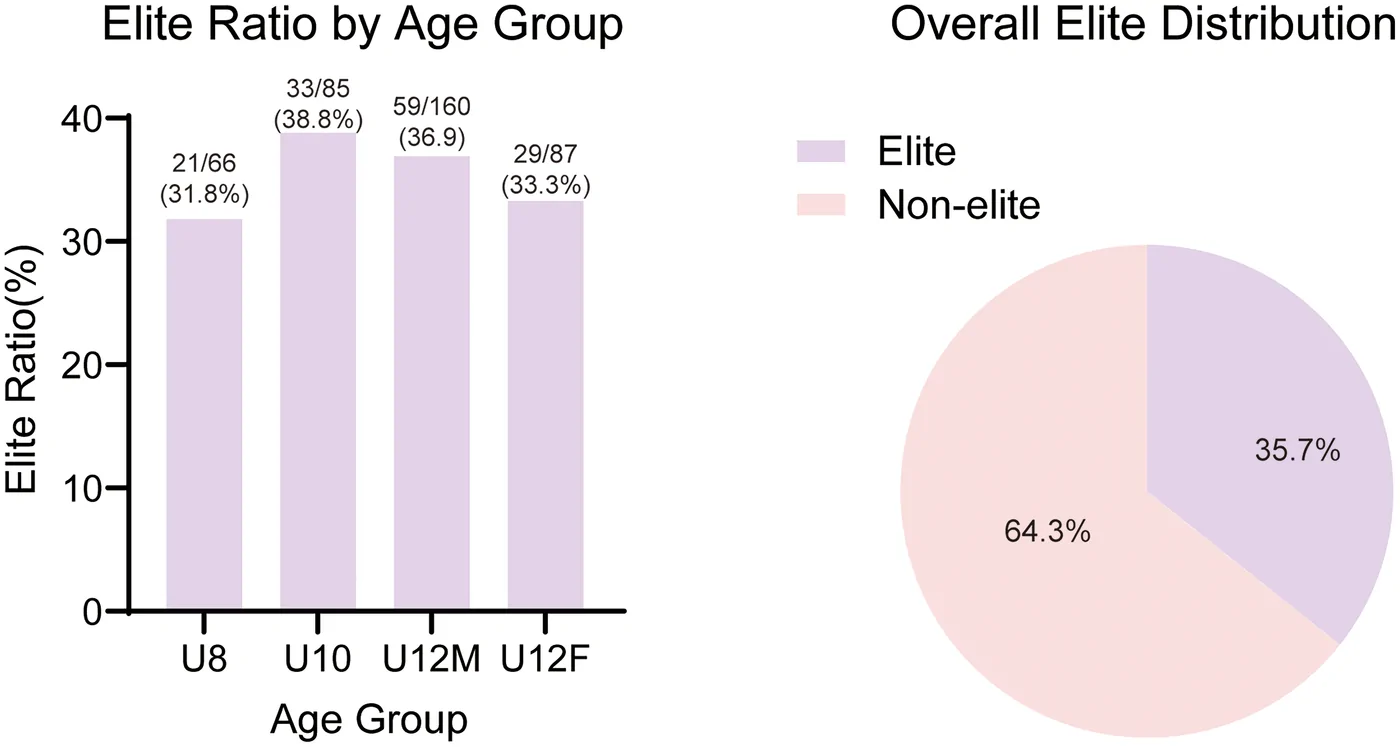
Elite ratio by age group and overall elite distribution.

Data were collected in early October 2024. The sample consisted of 398 Chinese children (311 males, 87 females), aged 7.0 to 12.9 years, from 20 schools and 34 regional teams, all participating in the Yunnan Football Association (YFA) Elite Training Camp and affiliated with the YFA, undergoing specialized football training and education. As shown in Figure 1, based on YFA expert coach evaluation and technical-tactical criteria during camp competition observations, 142 children were classified as “elite” (U8: *n* = 21; U10: *n* = 33; U12M: *n* = 59; U12F: *n* = 29), and 256 as “non-elite” (U8: *n* = 45; U10: *n* = 52; U12M: *n* = 101; U12F: *n* = 58). Inclusion criteria were: (1) age 7.0-12.9 years, having completed all tests with at least two recorded repetitions; (2) having previous participation in regional or school football leagues; (3) the absence of injuries or illnesses potentially affecting test performance. From a total of 429 players, 31 players were finally discarded for not meeting the inclusion criteria (see Section 2.5 for details). In early November 2024, 15 of the tested U12M athletes were subsequently selected for the Chinese Football Association's National Elite Youth Training Camp.

### Procedures

All objective and psychological tests were conducted by the School of Physical Education, Yunnan University, following standardized protocols. Subjective performance ratings were provided by YFA expert coaches based on match observations. All testing was completed at the Kunming Haigeng Sports Training Base between between October 1 and 6, 2024.Upon arrival at the testing site, participants' height and weight were measured. Next, and even though all the players were accustomed to the tests, because they had previously performed them during training, they were reminded of protocols before starting. Then, all subjects performed a general warm-up and a specific warm-up (including performing each of the tests as a pre-test).EPQ, which was always administered last, the order of the remaining tests was randomized. Sufficient recovery time (> 5 min) was ensured between tests. All objective tests were performed twice to ensure measurement reliability, with the best attempt recorded as the final score.

The tools and procedures we evaluated adhere to strict standards. Participants stood barefoot on an ultrasonic stadiometer while their height and weight were measured. Physical tests were recorded using infrared timers: 10-meter/30-meter sprint performance (U8/U10 groups: 10 m; U12 groups: 30 m). The device automatically captures data from the start beam and finish beam, and we recorded the best result from two attempts. The standing long jump was measured using a testing mat, recording the vertical distance from the takeoff line to the nearest landing spot. Dribbling speed was conducted on a route marked by cones. Completion times were also recorded by infrared timers. Football technical assessments included: a passing accuracy test, where participants made 5 passes through a 1.5-meter wide goal made of cones from a designated area within 10 s; the U12 groups additionally completed left-foot and right-foot shooting tests, where participants were required to strike the ball first-time into a target area within an eight-a-side goal from 15 m. For all physical and technical tests, athletes wore standardized bibs, and field layouts and distances were verified using a PVC fiberglass measuring tape. Psychological assessment was administered using the Junior Eysenck Personality Questionnaire, conducted independently after the physical tests, with scores for the Extraversion (E) and Neuroticism (N) scales extracted. Subjective performance evaluation was conducted by a team of expert coaches certified by the YFA. After observing players in match-play scenarios (small-sided games), and drawing on the Chinese Football Association's scouting evaluation indicators, the coaches independently rated players across four dimensions: MT, MP, MTA and MSO. All our testing equipment, such as infrared timers and long jump mats, are professional-grade and designed to minimize measurement errors as much as possible.

### Data analysis and modeling

Data from the originally collected 429 adolescent football players (U8, U10, U12M, U12F) were processed, and 31 records were appropriately excluded because of missing data, duplicates, or implausible values, leaving a final sample of 398 athletes. The data processing procedure was clearly and systematically carried out: feature standardization was applied only to the Logistic Regression model, as tree-based models (RF, XGBoost) are invariant to monotonic feature transformations, then one-hot encoding of the categorical variables (playing position, ethnicity), followed by data cleaning and BMI calculation, and median imputation for the remaining 3.1% of missing values by age group. Then performed exploratory data analysis separately for each age group, starting with descriptive statistics, followed by comparison of elite versus non-elite groups using independent t-tests with Welch's correction and Cohen's d effect size estimation, and finally examination of feature relationships via Spearman correlations. In the modeling stage, the data for each age group was first stratified into a training set (80%) and a held-out test set (20%), preserving the original proportion of elite to non-elite athletes. Within the training set only, a two-layer cross-validation framework was employed. Outer layer: a 5-fold stratified cross-validation was used to evaluate model stability, yielding the CV Accuracy and CV ROC-AUC. Inner layer: within each outer fold, hyperparameter tuning was performed using 5-fold stratified cross-validation — GridSearchCV for RF and LR, and RandomizedSearchCV for XGBoost. The final model was retrained on the entire training set using the optimal hyperparameters and evaluated once on the independent held-out test set to obtain test-set metrics (ROC-AUC, Accuracy, F1, Precision, Recall). This nested structure ensures no data leakage between parameter tuning and performance evaluation. Model interpretability was achieved through the SHAP analysis, employing TreeExplainer (with KernelExplainer as fallback) to compute global feature importance. For the U12F XGBoost model, where TreeExplainer was incompatible, KernelExplainer was used with a background dataset of 50 randomly sampled training instances (n samples=100) to approximate Shapley values. A dedicated validation analysis within the U12M cohort (*n* = 160) was conducted. This analysis quantified provincial-national selection consistency using Cohen's kappa, assessed model performance in retrospectively identifying the 15 national elites, and identified potentially underestimated players based on the following criterion: high model-predicted probability of being a national elite (≥0.7) but not selected at national level.

All analyses were implemented in Python 3.9 using scikit-learn, XGBoost, and SHAP, with full code provided to ensure reproducibility. To quantify estimation uncertainty, we estimated 95% confidence intervals (CIs) for ROC-AUC and F1-score using Bootstrap resampling (1,000 iterations). A Permutation Test (1,000 permutations) was conducted to verify whether model performance significantly exceeded random guessing (*p* < 0.05).

## Results

After data cleaning, 398 youth football players were included (U8: *n* = 66; U10: *n* = 85; U12M: *n* = 160; U12F: *n* = 87). The overall elite selection rate was 35.7% (142/398), with rates of 31.8%, 38.8%, 36.9%, and 33.3% for the U8, U10, U12M, and U12F groups, respectively, indicating a consistent but manageable class imbalance across cohorts.

As shown in Table 1, descriptive statistics revealed distinct anthropometric, physical, technical, tactical, and psychological profiles between elite and non—elite players across all age groups (U8, U10, U12M, U12F). Table 2 further statistically validated these observed differences, with t-tests confirming that all differences were statistically significant (all *p* ≤ 0.001) and effect sizes ranged from moderate to large (e.g., SP30 in U12M: d = −0.90; SLJ in U12M: d = 1.12). Technical skills (PAC, DRI, LFS/RFS for U12) also showed meaningful disparities, with elite players achieving higher PAC, quicker DRI, and more goals in shooting (LFS/RFS for U12), where *p*—values were mostly < 0.001 (except LFS in U12F, *p* = 0.074) and effect sizes were moderate to large (e.g., LFS in U12M: d = 1.08). For psychological and match—related metrics, EXT and NEU did not show consistent significant differences across groups, while MTA and MSO were significantly higher among elite players (MTA: *p* < 0.001 for U10, U12M, U12F; MSO: *p* < 0.01 for U8, *p* < 0.05 for U12M), with effect sizes ranging from small to moderate.

**Table 1:** Descriptive characteristics of participants by age group and elite status (mean ± SD).

Characteristic	U8 (*n* = 66)		U10 (*n* = 85)		U12M (*n* = 160)		U12F (*n* = 87)	
	Elite (*n* = 21)	Non-elite (*n* = 45)	Elite (*n* = 33)	Non-elite (*n* = 52)	Elite (*n* = 59)	Non-elite (*n* = 101)	Elite (*n* = 29)	Non-elite (*n* = 58)
HT (cm)	130.86 ± 3.80	128.20 ± 6.38	138.03 ± 6.89	137.75 ± 6.73	155.68 ± 9.31	149.98 ± 9.14	155.90 ± 4.90	151.97 ± 7.10
BM (kg)	25.68 ± 2.94	25.07 ± 5.44	30.24 ± 3.74	30.50 ± 6.96	45.00 ± 9.90	41.63 ± 10.26	46.85 ± 7.38	42.23 ± 8.12
BMI (kg/m^2^)	14.97 ± 1.24	15.14 ± 2.31	15.83 ± 1.17	15.92 ± 2.52	18.38 ± 2.44	18.38 ± 3.79	19.26 ± 2.81	18.14 ± 2.40
SP10 (s)	2.20 ± 0.08	2.31 ± 0.10	2.14 ± 0.08	2.23 ± 0.11	-	-	-	-
SP30 (s)	-	-	-	-	4.74 ± 0.30	5.06 ± 0.40	5.05 ± 0.28	5.26 ± 0.22
SLJ (m)	1.58 ± 0.07	1.46 ± 0.12	1.77 ± 0.12	1.66 ± 0.14	2.08 ± 0.20	1.87 ± 0.18	1.91 ± 0.12	1.76 ± 0.15
DRI (s)	12.94 ± 1.02	15.38 ± 2.09	12.51 ± 1.01	13.48 ± 1.45	11.51 ± 0.94	12.36 ± 1.61	13.04 ± 1.54	13.85 ± 1.12
PAC	2.62 ± 1.50	2.04 ± 1.19	3.24 ± 1.32	2.54 ± 1.20	2.25 ± 1.14	2.07 ± 1.05	2.90 ± 1.05	2.28 ± 1.21
LFS	-	-	-	-	2.51 ± 1.09	1.27 ± 1.22	0.89 ± 1.13	0.44 ± 0.95
RFS	-	-	-	-	2.88 ± 1.08	2.63 ± 0.94	2.45 ± 1.09	2.17 ± 1.45
EXT	9.67 ± 6.54	8.80 ± 5.97	8.61 ± 7.42	8.15 ± 7.83	8.73 ± 6.91	10.44 ± 6.58	11.34 ± 6.69	10.09 ± 8.39
NEU	−4.86 ± 6.94	−8.73 ± 9.35	−13.33 ± 6.89	−9.94 ± 9.90	−10.51 ± 8.31	−11.05 ± 9.26	−7.41 ± 10.98	−9.00 ± 10.87
MT	22.57 ± 5.71	19.62 ± 4.65	21.45 ± 7.50	20.08 ± 6.23	23.24 ± 4.15	19.21 ± 5.13	24.62 ± 2.66	19.55 ± 4.22
MP	15.38 ± 2.16	13.82 ± 2.47	13.70 ± 4.24	13.04 ± 3.85	15.66 ± 3.34	13.09 ± 3.48	17.24 ± 2.59	12.03 ± 3.36
MTA	17.05 ± 3.09	16.13 ± 3.02	18.85 ± 4.22	15.92 ± 3.60	19.27 ± 2.85	17.15 ± 3.75	18.10 ± 2.69	14.69 ± 2.87)
MSO	8.33 ± 0.66	9.31 ± 1.73	7.42 ± 2.26	7.69 ± 2.23	9.49 ± 1.10	10.62 ± 4.59	9.14 ± 1.48	8.69 ± 1.51

HT, height; BM, body mass; BMI, body mass index; SP10, 10-meter sprint; SP30, 30-meter sprint; SLJ, standing long jump; DRI, dribbling speed; PAC, passing accuracy; LFS, left-foot shooting; RFS, right-foot shooting; EXT, Extraversion; NEU, Neuroticism; MT, match technical; MP, match physical; MTA, match tactical; MSO, match social. Dash (-) indicates test not administered for that age group.

**Table 2:** Results of t-tests comparing elite and non-elite players on key continuous variables within age groups.

Variable	Age group	*t*	*p*	*d*
SP10 (s)	U8	−4.96	<.001	−1.24
	U10	−4.16	<.001	−0.89
	U12M	-	-	-
	U12F	-	-	-
SP30 (s)	U8	-	-	-
	U10	-	-	-
	U12M	−5.68	<.001	−0.90
	U12F	−3.50	.001	−0.83
SLJ (m)	U8	5.22	<.001	1.25
	U10	3.84	<.001	0.84
	U12M	6.76	<.001	1.12
	U12F	5.15	<.001	1.12
DRI (s)	U8	−6.38	<.001	−0.78
	U10	−3.65	<.001	−0.64
	U12M	−4.17	<.001	−0.64
	U12F	−2.52	.015	−0.60
LFS	U8	-	-	-
	U10	-	-	-
	U12M	6.66	<.001	1.08
	U12F	1.83	.074	0.44
EXT	U8	0.52	.609	0.14
	U10	0.27	.790	0.06
	U12M	−1.53	.128	−0.25
	U12F	0.76	.451	0.17
NEU	U8	1.88	.065	0.47
	U10	−1.86	.067	−0.40
	U12M	0.38	.704	0.06
	U12F	0.64	.526	0.15
MTA	U8	1.13	.266	0.30
	U10	3.29	.002	0.75
	U12M	4.04	<.001	0.64
	U12F	5.45	<.001	1.23
MSO	U8	−3.31	.002	−0.75
	U10	−0.54	.594	−0.12
	U12M	−2.36	.020	−0.34
	U12F	1.32	.192	0.30

SP10, 10-meter sprint; SP30, 30-meter sprint; SLJ, standing long jump; DRI, dribbling speed; LFS, left-foot shooting; EXT, Extraversion; NEU, Neuroticism; MTA, match tactical; MSO, match social. T-test (elite vs. non-elite): Statistical significance was set at *p* < 0.05. d: Cohen's d effect size (positive values indicate elite > non-elite for that variable). Dash (-) indicates test not administered for that age group.

Following the univariate comparisons, we employed machine learning to model the multivariate prediction of elite status. We found distinct predictive patterns across age groups and genders. From the results in Table 3 it is very clearly established that the RF model performed best for the U8, U10, and U12M groups, obtaining test-set ROC-AUC values of 0. 900, 0. 957, and 0. 954, respectively, whereas the XGBoost model was optimal for the U12F group with a test-set ROC-AUC of 0. 944. More importantly, all models exhibited excellent generalization, since the cross-validation ROC-AUC scores ranged from 0. 804 (U10)–0. 931 (U12F). Test-set accuracy was above 84% in every case. Bootstrap-estimated 95% CIs for ROC-AUC ranged from 0.644–1.000 across groups, reflecting acceptable uncertainty given the small test sets. Permutation Tests confirmed that all best models significantly outperformed random guessing (all *p* < 0.05). An especially interesting and useful point is the different trade-offs between sensitivity and specificity: the U10 RF model had perfect precision (1. 000) but only a recall of 0. 714, while the U12F XGBoost model achieved a nicely balanced 0. 833 for both.

**Table 3:** Performance of the best machine learning model per age group on the independent test set.

Age group	Best model	ROC-AUC (95% CI)	Accuracy	F1-Score(95% CI)	Precision	Recall	CV Accuracy (mean ± SD)	CV ROC-AUC (mean ± SD)	Permutation *p*-value
U8	RF	0.900 (0.644–1.000)	0.857	0.784 (0.400–1.000)	0.667	1.000	0.877 ± 0.078	0.978 ± 0.027	0.011
U10	RF	0.957 (0.818–1.000)	0.882	0.812 (0.500–1.000)	1.000	0.714	0.718 ± 0.069	0.804 ± 0.073	0.001
U12M	RF	0.954 (0.875–1.000)	0.844	0.793 (0.588–0.952)	0.769	0.833	0.856 ± 0.042	0.897 ± 0.048	0.001
U12F	XGBoost	0.944 (0.740–1.000)	0.889	0.823 (0.500–1.000)	0.833	0.833	0.840 ± 0.054	0.931 ± 0.038	0.002

The optimal model for each age group was selected based on the highest ROC-AUC score on the independent test set. ROC-AUC (95% CI) and F1-Score (95% CI) were estimated via Bootstrap resampling (1,000 iterations). Permutation *p*-value indicates whether the model's AUC significantly exceeds random guessing (1,000 permutations); *p* < 0.05 denotes statistical significance. All metrics were calculated on the held-out test set (U8: *n* = 14; U10: *n* = 17; U12M: *n* = 32; U12F: *n* = 18).

The predictions of the models were analyzed using SHAP analysis, and from Table 4 it is clearly and neatly seen that DRI was the strongest predictor in both the U8 (SHAP: 0.104) and U10 (0.095) groups. For older players, however, the important predictors changed: LFS was most important for U12M (0.083), whereas MTA was the key predictor for U12F (0.107). Psychological traits (EXT, NEU) had consistently low importance ranks.

**Table 4:** Top 5 most important features for predicting provincial elite status, ranked by mean absolute SHAP value per age group.

Rank	U8 (RF)	U10 (RF)	U12M (RF)	U12F (XGBoost)
1	DRI (0.104)	DRI (0.095)	LFS (0.083)	MTA (0.106)
2	SLJ (0.098)	SP10 (0.069)	DRI (0.074)	SLJ (0.080)
3	SP10 (0.038)	SLJ (0.062)	SLJ (0.052)	DRI (0.070)
4	MSO (0.035)	MTA (0.038)	MTA (0.049)	MP (0.062)
5	HT (0.034)	MSO (0.035)	SP30 (0.035)	BMI (0.052)

DRI, dribbling speed; SLJ, standing long jump; SP10, 10-meter sprint; MSO, match social; HT, height; MTA, match tactical; LFS, left-foot shooting; SP30, 30-meter sprint; MP, match physical; BMI, body mass index. Values in parentheses represent the mean absolute SHAP value.

**Figure 2:**
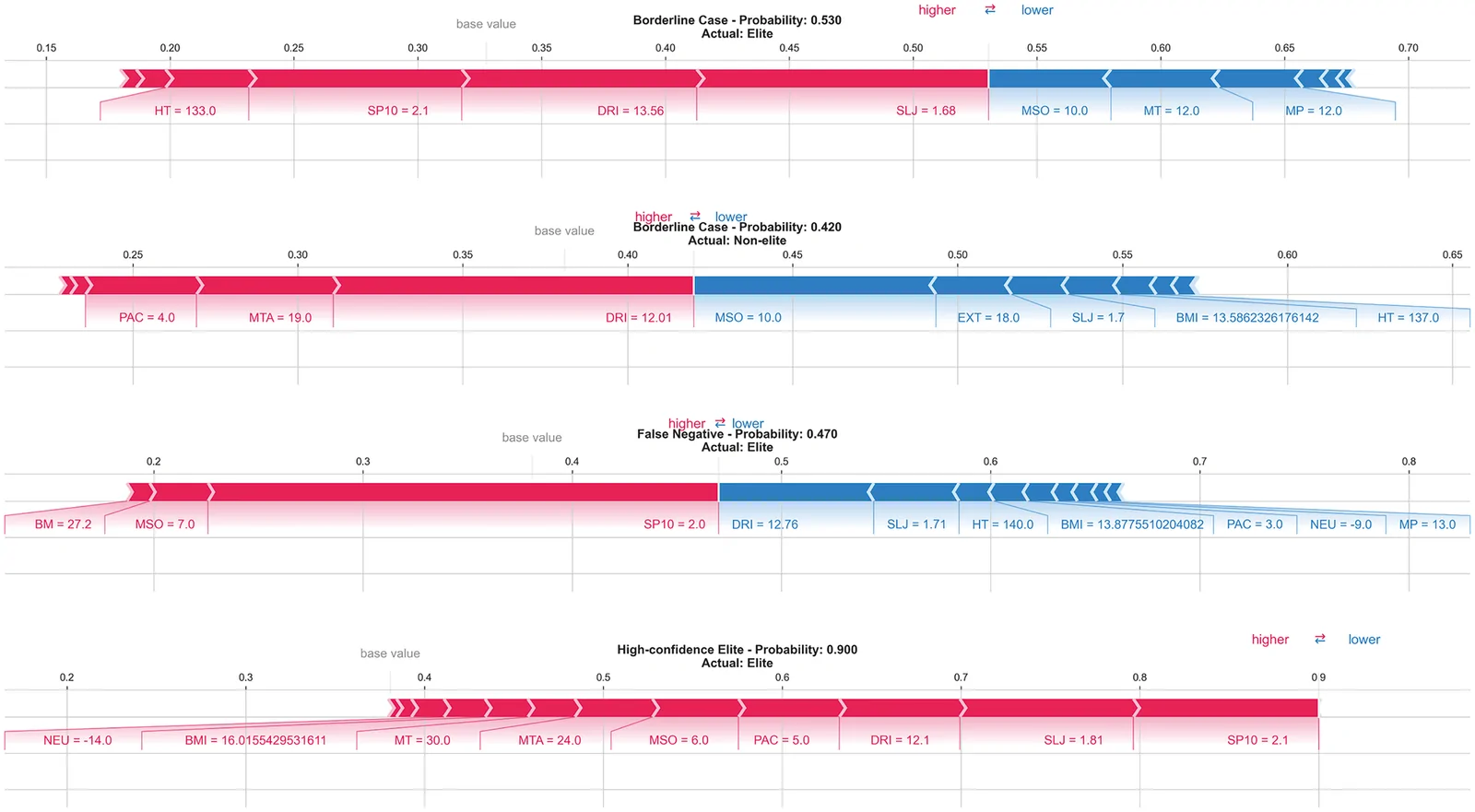
SHAP force plot illustrating feature contributions to elite classification probability for representative cases across age groups.

To move beyond global feature importance and elucidate the decision logic for individual players, we generated SHAP force plots for representative cases across different age groups and prediction scenarios (Figure 2), highlighting three distinct patterns of feature contribution. (1) Borderline cases (predicted probability∼0.4-0.55), where balanced positive and negative feature contributions led to uncertain predictions. For example, a U8 player (probability = 0.530) whose prediction was primarily driven by positive contributions from SLJ (1.68 m), SP10 (2.1 s), and DRI (13.56 s), and a U12F player (probability = 0.420) where PAC (4.0) and MTA (19.0) were key positive contributors. (2) False negative cases (actual elite, predicted probability < 0.5), revealing how strong positive contributions from some features [e.g., a U8 player's SP10 (2.0 s) and MSO (7.0)] could be offset by strong negative drivers [e.g., DRI (12.76 s)], preventing the prediction from crossing the elite threshold. (3) High-confidence elite cases (predicted probability ≥0.8), such as a U10 player (probability = 0.900), where overwhelmingly positive contributions from key features like SLJ (2.20 m), MTA (18.0), and SP30 (4.27 s) resulted in minimal negative influence.

**Figure 3:**
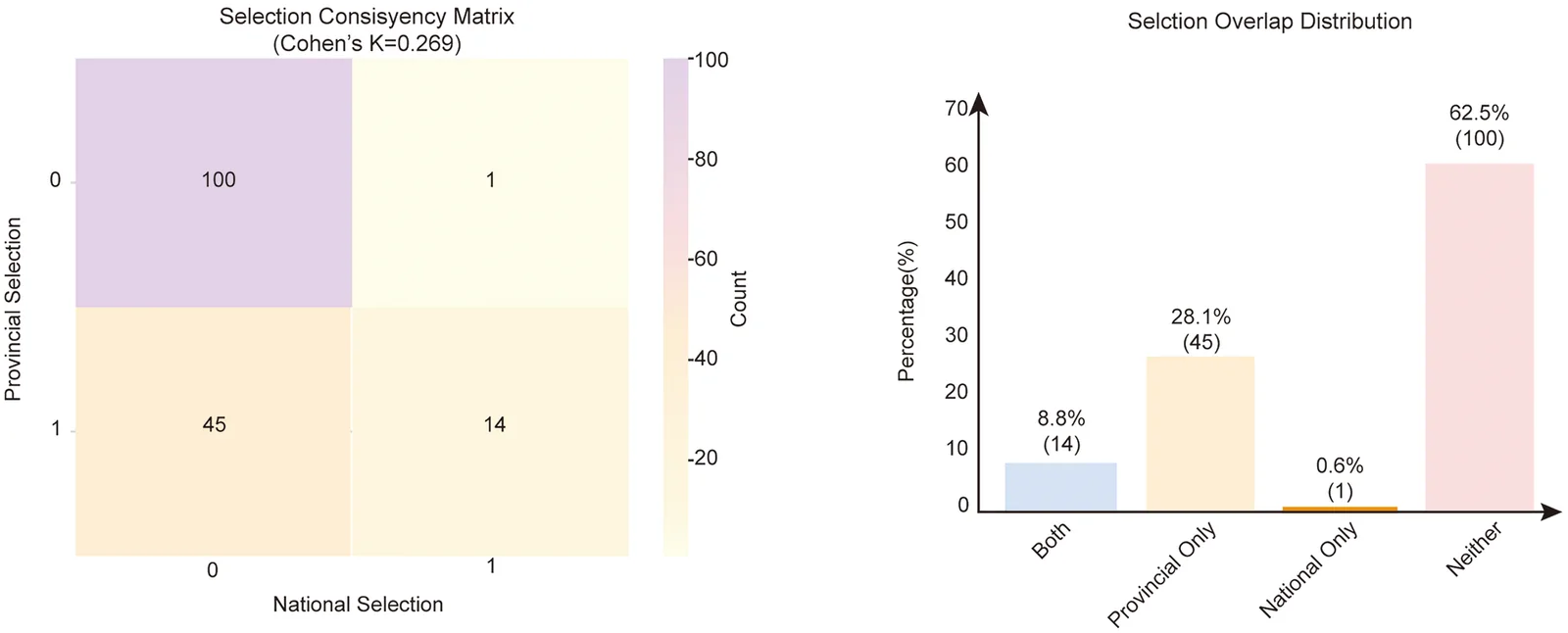
Consistency between provincial and national elite selection in the U12M group (*n* = 160).

To validate the relevance of provincial selection model, we examined its consistency with higher-level selection outcomes in the U12M age group. In this group, 15 players were subsequently selected as national elites by the Chinese Football Association. Figure 3 shows the confusion matrix between the two selection levels, showing significant overlap. 14 out of the 15 national elite players (93.3%) were also selected by the provincial selection, while 44 provincial elite players were not selected at the national level. We quantified the consistency between these two selection levels using Cohen's kappa, which yielded a fair result (*K* = 0.269).

**Figure 4:**
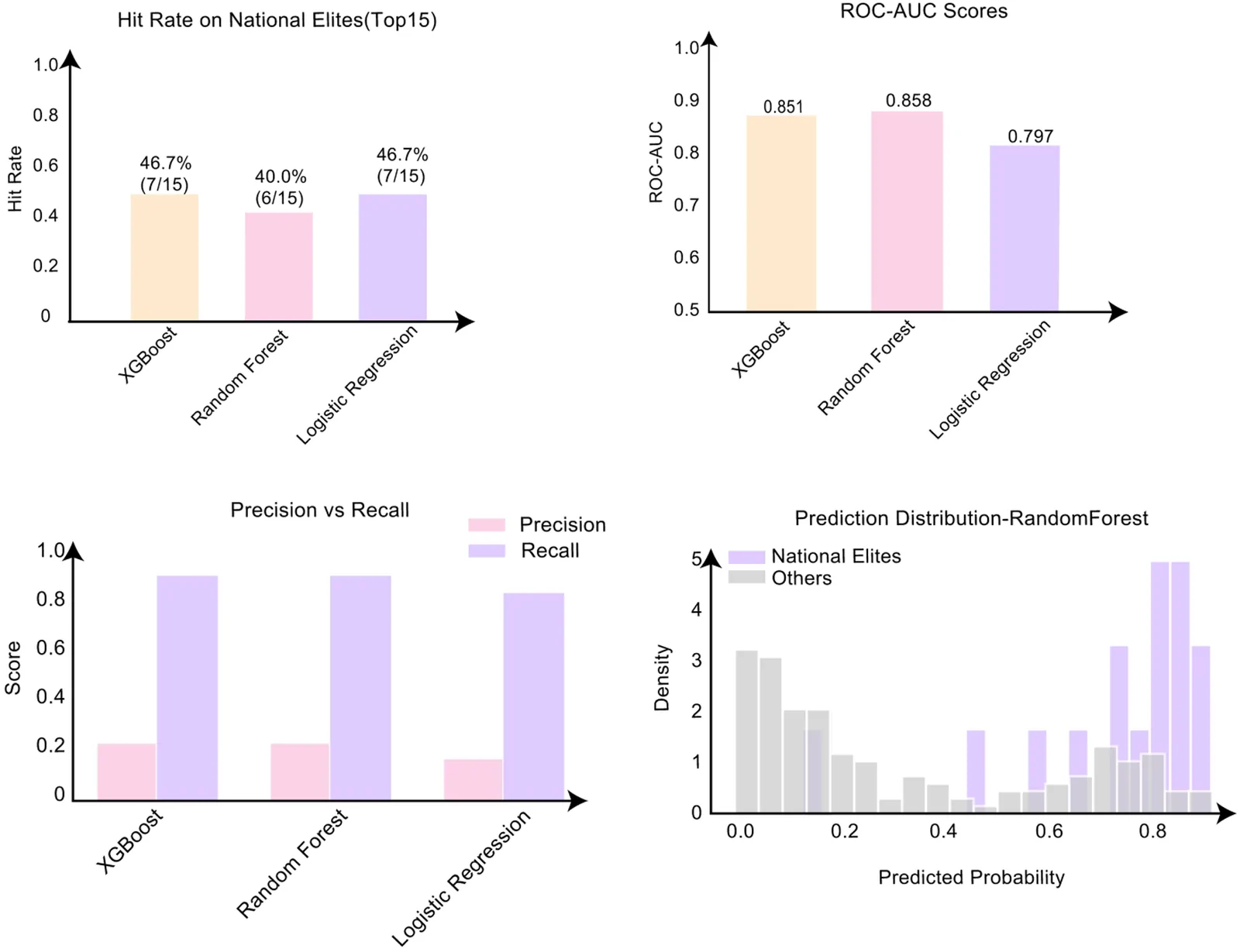
Performance evaluation of three machine learning models (XGBoost, RF, logisticRegression) for predicting national elite status among U12M players using the top 15 features selected by RF. (Upper left panel: Hit rate of national elites within the top 15 predicted candidates (percentage of true national elites identified among the top 15 highest-probability predictions). Upper right panel: Receiver Operating Characteristic (ROC) curve-derived AUC scores for each model. Lower left panel: Precision vs. Recall scores for each model (Precision=proportion of predicted elites that are true elites; Recall=proportion of true elites correctly identified). Lower right panel: Predicted probability distribution for national elite status using the RF model, with separate density plots for national elites (red) and non-elites (gray).).

We applied the U12 RF model, trained based on provincial selection results, to score the probability of all U12M players becoming national elites. In a retrospective evaluation of 15 known national elite players, we found that 6 of the 15 highest probability players predicted by the model were national elites, corresponding to a hit rate of 40.0% (Figure 4, upper left panel); notably, the XGBoost model achieved a slightly higher hit rate of 46.7% (7/15) under the same selection criterion. In parallel, the RF model demonstrated robust discriminative capacity for national elite status, achieving an ROC-AUC of 0.858 (Figure 4**,** upper right panel), while XGBoost yielded a comparable ROC-AUC of 0.851 and LR 0.797. Our evaluation of the precision-recall curves for the three models revealed that recall was consistently and significantly higher than precision. Both RF and XGBoost achieved a recall of 0.867 and a precision of 0.224 (Figure 4, lower left panel). The prediction distribution for the RF model (Figure 4, lower right panel) present a right-skewed probability density, with the 15 national elite players concentrated in the high-probability tail (predicted probability > 0.6).

To understand the characteristics that might distinguish the highest level of selection, we compared the 15 National Elites with the 44 players who were selected as Provincial Elites but not as National Elites (Table 5). Independent samples t-tests revealed that PAC was the only variable showing a statistically significant difference between the two groups (*p* = 0.003), with National Elites achieving higher scores. The effect size for this difference was large (*d* = 0.97). No other variable, including physical performance (SLJ, SP30), other technical skills (DRI, LFS), or coach ratings (MT, MP, MSO), showed a significant difference (*p* > 0.05 for all), with effect sizes ranging from negligible to small.

**Table 5:** Anthropometric and performance characteristics by elite Status in national elites (*n* = 15) and only provincial elites (*n* = 44).

Feature	National elites (*n* = 15)	Provincial elites (*n* = 44)	*t*	*p*	*d*
HT	159.80 ± 10.76	154.13 ± 8.40	1.86	0.078	0.59
BM	49.75 ± 13.27	43.68 ± 8.17	1.67	0.113	0.55
PAC	3.00 ± 1.00	2.00 ± 1.07	3.30	0.003	0.97
SLJ(m)	2.14 ± 0.22	2.05 ± 0.20	1.47	0.155	0.45
DRI(s)	11.37 ± 0.99	11.56 ± 0.92	−0.65	0.523	−0.20
SP30(s)	4.69 ± 0.35	4.77 ± 0.30	−0.83	0.415	−0.26
LFS	2.53 ± 1.30	2.49 ± 1.01	0.12	0.905	0.04
EXT	−10.60 ± 10.94	−10.49 ± 7.28	−0.04	0.971	−0.01
MT	24.53 ± 4.49	22.67 ± 4.03	1.43	0.167	0.44
MP	16.20 ± 2.51	15.40 ± 3.58	0.95	0.348	0.26
MSO	10.40 ± 3.31	9.47 ± 1.18	1.07	0.302	0.38

HT, height; BM, body mass; BMI, body mass index; SP30, 30-meter sprint; SLJ, standing long jump; DRI, dribbling speed; PAC, passing accuracy; LFS, left-foot shooting; EXT, Extraversion; MT, match technical; MP, match physical; MSO, match social. Data are presented as mean ± standard deviation (SD). *p* indicate independent samples t-test results comparing national elites with provincial elites. d: Cohen's d effect size (positive values indicate elite > non-elite for that variable).

Finally, in the U12M cohort, a total of 15 players were identified as potentially undervalued (predicted probability of being a national elite ≥ 0.7, but not selected at national level). In Table 6, we present the characteristics of the five players with the highest predicted probabilities. Notably, all five players were provincial elites. We found that their common feature is possessing at least one standout attribute (e.g., very fast DRI of 10.52 s in Player 1, or excellent SLJ of 2.42 m in Player 3). Other performance metrics are good but do not reach the top level. For instance, Player 1 has the highest predicted probability (0.946) and shows strong tactical rating (MTA = 20) and good speed (SP30 = 4.35 s), but a relatively modest left-foot shooting score.

**Table 6:** Characteristics of the top 5 potentially underestimated U12M players (predicted probability ≥ 0.7, not nationally selected).

Rank	Pred. Prob.	Prow. Selected	Nat. Selected	DRI(s)	SLJ(m)	SP30(s)	LFS	MTA
1	0.946	TRUE	FALSE	10.52	2.11	4.35	3	20
2	0.943	TRUE	FALSE	12.13	2.26	4.66	3	24
3	0.912	TRUE	FALSE	12.09	2.42	4.80	2	20
4	0.873	TRUE	FALSE	12.83	2.31	4.37	4	23
5	0.869	TRUE	FALSE	11.07	2.07	5.13	3	20

DRI, dribbling speed; SLJ, standing long jump; SP30, 30-meter sprint; LFS, left-foot shooting; MTA, match tactical. Pred. Prob.=Model-predicted probability of being a national elite. Prov./Nat. Selected=selection outcome at provincial/national level.

## Discussion

Our research findings empirically confirm that elite adolescent football status cannot be reduced to a single domain (1, 14). A model integrating anthropometric, physical, technical, psychological, and match performance demonstrates high predictive accuracy, aligning with the consensus on the complex interactions of talent (15). More importantly, through age-stratified SHAP analysis, we revealed how the importance of these dimensions differs across age groups. However, it should be noted that these apparent shifts partly reflect differences in the test metrics administered to each age group, rather than exclusively representing developmental transitions. For the U8 and U10 players, fundamental physiological abilities such as standing long jump and sprint speed, along with basic skills like dribbling, are the strongest predictors of elite status. Dribbling, as a foundational skill, provides support for subsequent specialized development (10, 16). Successfully dribbling under pressure depends on the integration of coordination, agility, and ball control. Our cross-sectional data consistently showed dribbling as an important predictor across all age groups, suggesting its fundamental role in differentiating elite status at multiple developmental stages. However, longitudinal tracking is needed to determine whether this reflects a causal developmental mechanism or a stable selection criterion (17, 18). For U12 players, the situation changes. However, U12 players present a distinct case: the key distinguishing factors become more specific. For U12F, cognitive attributes, namely their tactical understanding during matches, are the most important factor in distinguishing elite players. For U12M, skills with the non-dominant foot (left foot) are the primary differentiator. This makes perfect sense in a game context where defenders frequently attempt to push attackers toward their weaker side, so bilateral foot ability makes players harder to predict and gives them more viable options, which is undeniably a competitive advantage (19). Our finding empirically validates the consensus in football regarding the tactical value of bilateral skills (20). Thus, prioritizing the assessment and targeted training of non-dominant foot proficiency should be recognized as a high-return investment in youth talent development systems. Conversely, coach-rated cognitive attributes (tactical and physical understanding) dominate in the U12F group, indicating a developmental transition point. Our cross-sectional observation that tactical awareness was the strongest predictor in U12F, but not in younger groups, is consistent with developmental models such as Long-Term Athlete Development (LTAD), which posit a shift from general skills to sport-specific and cognitive abilities (21, 22). However, this pattern may reflect cohort differences rather than true developmental progression, and longitudinal studies are required to confirm such a shift (16). Therefore, talent identification programs should dynamically adjust based on the evolution of relevant factors across developmental stages. Our results collectively indicate that uniform, age-agnostic assessment protocols are suboptimal (23). The observed age-related differences in feature importance must be interpreted cautiously, as they are partially confounded by age-specific test batteries. Standardized assessments across age groups are needed to confirm true developmental trends.

However, not all theoretical expectations were supported by our data. The psychological constructs measured in our study—EXT and NEU from the EPQ, showed marked differences from theoretical expectations in the talent identification literature (14, 24). In most comparisons, statistically significant differences were not observed between the elite and non-elite groups (Table 2: all *p* > 0.05 for EXT; NEU *p* = 0.065 in U8, *p* = 0.067 in U10). Additionally, their predictive importance remained low in the SHAP analysis. For example, the SHAP values for NEU were more than three times lower than those of the most important predictor in each cohort. In terms of feature importance ranking, it ranked 7th in the U8 group, 6th in the U10 group, and did not make the top 10 in either of the two U12 groups. The consistency between statistical methods and interpretable machine learning warrants a careful, multi-faceted interpretation. First, it may indicate an “observable performance bias” within the provincial selection paradigm in China. In time-constrained assessment camps, evaluators may disproportionately prioritize immediately demonstrable physical and technical skills over latent psychological traits, a tendency documented in traditional selection settings (15, 25). The model, trained to replicate this selection outcome, consequently learns to prioritize the same features. Second, the broad, domain-general dimensions of the EPQ may lack the specificity to capture the psychological constructs most consequential for football performance, such as sport-specific grit or perceptual-cognitive skills under pressure (26, 27). As noted in critiques of personality testing in sports (28), traits like conscientiousness or specific facets of motivation often show stronger associations with athletic performance than the broader dimensions of extraversion or neuroticism. Third, the cross-sectional design of our study inherently limits causal inference. The minimal SHAP contribution does not preclude psychological attributes from influencing long-term talent development trajectories. The models were optimized to predict a single time-point selection outcome; traits that exert their effect cumulatively over time—moderating responses to training, adversity, or competition (29, 30) — may not yield a strong SHAP signal in a cross-sectional snapshot, despite their theoretical importance for talent development. Our findings suggest that generic personality assessments cannot be directly integrated into talent identification processes. We advocate for the development and validation of measurement tools for psychological skills tailored to football-specific contexts in the future.

Our second key finding reveals the practical limitations of subjective, expert-driven talent identification (2, 31). While provincial systems can serve as effective initial screening tools, capturing 93% of national-level elite talent, but there is only a fair degree of consistency between provincial and national selections (*K* = 0.269), highlighting significant discrepancies. The 44 players selected provincially but not advancing to the national level, along with 1 national elite talent overlooked by the provincial system, indicate differences in assessment criteria and thresholds. National selectors likely see more players and may prioritize different combinations of attributes (14, 32). Given this, we see our provincially trained model as providing a useful second opinion. It flags players with high predicted potential who were not chosen nationally. Tracking how these particular players develop will be the real test of our model's accuracy. It could also reveal talent that momentary dips in form or subjective biases might have hidden (9, 17, 33). Therefore, we believe the value of this study lies in providing a data-driven framework to assist coaches in identifying youth football talent. These models as decision-making tools, offer additional perspectives to help mitigate personal biases (9, 34, 35). Our framework has three specific applications: (1) Using SHAP, the most relevant age-related indicators (such as dribbling speed or standing long jump) can be identified, optimizing the assessment process for more efficient testing (25). (2) The models can flag players who might otherwise be overlooked. Coaches re-evaluating these ‘underrated’ individuals may identify candidates whose current performance does not fully reflect their potential (36), though whether this represents later-developing talent or transient advantages requires longitudinal validation (24). (3) SHAP can translate prediction results into personalized feedback. By showing how specific attributes influence assessment outcomes, coaches can gain a clearer understanding of the rationale behind their developmental decisions and implement more targeted interventions (8, 37).

From a methodological perspective, we argue that this study establishes a transparent and reproducible ML workflow. This makes a very clear and useful contribution to bringing data science into youth sports by fully documenting the workflow from data cleaning, age-specific preprocessing, model tuning, to interpretation (38). More importantly, it identifies explicit trade-offs in model choice: RF dominated the U8, U10, and U12M groups (ROC-AUC:0. 900–0. 954), which is explained by its robustness to small samples and imbalanced classes (39). Because the method is insensitive to feature scaling, it is naturally and nicely suited for the heterogeneous metrics that are typical in multi-dimensional sports assessment (39). By contrast, XGBoost performed best for U12F players (ROC-AUC: 0. 944), probably because its boosting architecture is exceptionally good at learning subtle non-linear relationships in coach ratings, such as tactical awareness (MTA, SHAP = 0. 107) (8). Our work also shows clearly that the necessity of age-stratified modeling when evaluation criteria differ across developmental stages is a common but frequently neglected issue in longitudinal sports research (40). The high AUC values reported are practically very meaningful: a model with AUC 0.90 has a 90% chance of correctly ranking an elite athlete above a non-elite player, hence it clears the bar for a“useful”test in sport science (AUC  >  0.75). While LR performed adequately, Tree-based ensembles (RF, XGBoost) consistently matched or beat the baseline method, therefore their value in this context is clearly established. More importantly, SHAP provides both global and local explanations, (8) making it a natural way to open the“black box”of ensemble models (41). However, there is a definite technical limitation: TreeExplainer failed on the U12F XGBoost model, so we were forced to use the slower KernelExplainer. Hence, there is a clear trade-off between predictive performance and interpretability in resource—constrained youth sports environments (7, 42). On the ethics side, we must stress that algorithms trained on past selections inevitably bake in human biases, so if coaches historically undervalued mental toughness, the model will learn to ignore that trait, thus narrowing what “talent” means (43, 44). Therefore, we argue that such tools should only support, not replace, coaches' judgment, and advocate for a Human-in-the-Loop (HITL) approach (45, 46). Coaches can blend model outputs with their own observations. Finally, testing the models against national selections provided a rigorous form of external validation, going beyond mere internal statistical checks to assess how well predictions hold up against a higher standard.

Our study's conclusions should be considered in light of methodological limitations. First, although the overall sample size of our experiment (*n* = 398) is relatively large for adolescent populations specific to particular sports, the statistical power remains limited due to smaller test sample sizes in some age groups (U8, *n* = 14; U10, *n* = 17) (47). A notable limitation of this study is the inability to account for Relative Age Effects (RAE), a well-documented confounding factor in youth talent identification (40). RAE refers to the systematic advantage enjoyed by athletes born earlier in the selection year, who tend to be physically more mature and thus more likely to be identified as “talented” by coaches (48). In our dataset, precise birth dates were unavailable, precluding the calculation of relative age quartiles or the inclusion of birth quarter as a covariate in the models. Consequently, the predictive importance attributed to anthropometric and physical fitness metrics—such as standing long jump, sprint speed, and dribbling speed—may be partially inflated by the underlying developmental advantages conferred by older relative age within each age band, rather than solely reflecting intrinsic athletic talent (49). This is particularly relevant for the U8 and U10 groups, where physical attributes were among the strongest predictors (Table 4). Future studies should collect birth date data and explicitly control for RAE, for example by including birth quarter as a covariate or stratifying analyses by relative age quartiles. Such adjustments would allow a cleaner separation of genuine talent signals from age-related developmental noise. Additionally, the test batteries differed across age groups: U8/U10 completed a 10-meter sprint, while U12 groups completed a 30-meter sprint and left/right-foot shooting tests. Consequently, some observed shifts in feature importance—such as the prominence of left-foot shooting in U12M—reflect differences in available test metrics rather than purely developmental changes. Future studies should adopt age-consistent test batteries to disentangle developmental trends from measurement artifacts (24). Second, our cross-sectional study design limits causal inference (15). Features identified in the study as associated with elite level at a single time point can predict it, but we cannot distinguish correlation from causation or reveal dynamic developmental pathways (50). For example, while dribbling performance is consistently an important predictor, it is unclear whether it represents a stable trait (51), a result of specific training (52, 53), or an indicator of other potential factors that change with growth and maturation (54). Longitudinal studies are needed to determine which early predictors maintain their predictive validity over time and how the relative importance of physical, technical, and cognitive attributes changes during puberty. We recommend shifting from talent identification at a single time point to developmental trajectory modeling, which is crucial for evidence-based talent development (55).

Our study has several limitations that point to clear directions for future work. First, the reliance on subjective coach ratings means that more objective, detailed performance data such as high-speed running from GPS (56), or tactical behaviors captured by instrumented video tracking could provide a richer picture (57). Second, while the EPQ gave us a broad personality profile, constructs like grit or growth mindset might be more directly relevant to long-term development in adolescents (58). Since the present study is rooted in the specific context of youth football in Yunnan Province, China, the focus on technical skills such as dribbling is likely a reflection of local coaching philosophy (59). Hence, putting greater emphasis on technical attributes in youth training may not be directly applicable to other environments (60). The talent indicators derived in this study must be validated across different contexts (61). Sweeney et al. (2023) have clearly shown that talent systems have local biases, such as a preference for early maturers (4), it follows logically that future studies should validate the present model in other Chinese provinces and, even better, in regions with different football cultures (e.g., Germany or Brazil) (62). Only through such comparisons can reliably distinguish universal predictors of football talent from those influenced by the local environment.

## Data Availability

The original contributions presented in the study are included in the article/Supplementary Material, further inquiries can be directed to the corresponding author.
