# Supplementary material for: Explainable machine learning for age-specific talent identification in Chinese youth football
Source: Front Sports Act Living. 2026 Sep 2;8:1912796. doi: 10.3389/fspor.2026.1912796 (PMC13579789; doi:10.3389/fspor.2026.1912796)
Supplement: Supplementary file 1 [file Datasheet1.pdf]

Table. Hyperparameter Search Ranges and Optimal Configurations.

Age Group	Model	Parameter	Search Range	Optimal Value
U8	Random Forest	n_estimators	[100, 200, 300, 500]	500
		max_depth	[5, 10, 15, 20, None]	15
		min_samples_split	[2, 5, 10, 15]	5
		min_samples_leaf	[1, 2, 4, 6]	2
		max_features	['sqrt', 'log2', None]	log2
		bootstrap	[True, False]	True
		class_weight	['balanced', 'balanced_subsample', None]	balanced_subsample
	XGBoost	n_estimators	[100, 200, 300, 500]	200
		max_depth	[3, 5, 7, 9, 11]	7
		learning_rate	[0.01, 0.05, 0.1, 0.2]	0.1
		subsample	[0.6, 0.7, 0.8, 0.9, 1.0]	0.6
		colsample_bytree	[0.6, 0.7, 0.8, 0.9, 1.0]	0.6
		gamma	[0, 0.1, 0.2, 0.3]	0.1
		reg_alpha	[0, 0.1, 0.5, 1.0]	0.5
		reg_lambda	[0.5, 1.0, 1.5, 2.0]	1.5
		min_child_weight	[1, 3, 5, 7]	1
U10	Random Forest	n_estimators	[100, 200, 300, 500]	200
		max_depth	[5, 10, 15, 20, None]	15
		min_samples_split	[2, 5, 10, 15]	2
		min_samples_leaf	[1, 2, 4, 6]	1
		max_features	['sqrt', 'log2', None]	None
		bootstrap	[True, False]	True
		class_weight	['balanced', 'balanced_subsample', None]	balanced_subsample
	XGBoost	n_estimators	[100, 200, 300, 500]	500
		max_depth	[3, 5, 7, 9, 11]	11
		learning_rate	[0.01, 0.05, 0.1, 0.2]	0.1
		subsample	[0.6, 0.7, 0.8, 0.9, 1.0]	0.8
		colsample_bytree	[0.6, 0.7, 0.8, 0.9, 1.0]	0.7

		gamma	[0, 0.1, 0.2, 0.3]	0.1
		reg_alpha	[0, 0.1, 0.5, 1.0]	1.0
		reg_lambda	[0.5, 1.0, 1.5, 2.0]	1.0
		min_child_weight	[1, 3, 5, 7]	5
U12M	Random Forest	n_estimators	[100, 200, 300, 500]	500
		max_depth	[5, 10, 15, 20, None]	15
		min_samples_split	[2, 5, 10, 15]	5
		min_samples_leaf	[1, 2, 4, 6]	2
		max_features	['sqrt', 'log2', None]	log2
		bootstrap	[True, False]	True
		class_weight	['balanced', 'balanced_subsample', None]	balanced_subsample
	XGBoost	n_estimators	[100, 200, 300, 500]	100
		max_depth	[3, 5, 7, 9, 11]	7
		learning_rate	[0.01, 0.05, 0.1, 0.2]	0.05
		subsample	[0.6, 0.7, 0.8, 0.9, 1.0]	0.8
		colsample_bytree	[0.6, 0.7, 0.8, 0.9, 1.0]	0.7
		gamma	[0, 0.1, 0.2, 0.3]	0.1
		reg_alpha	[0, 0.1, 0.5, 1.0]	0.5
		reg_lambda	[0.5, 1.0, 1.5, 2.0]	2.0
		min_child_weight	[1, 3, 5, 7]	3
U12F	Random Forest	n_estimators	[100, 200, 300, 500]	300
		max_depth	[5, 10, 15, 20, None]	15
		min_samples_split	[2, 5, 10, 15]	2
		min_samples_leaf	[1, 2, 4, 6]	2
		max_features	['sqrt', 'log2', None]	log2
		bootstrap	[True, False]	True
		class_weight	['balanced', 'balanced_subsample', None]	balanced
	XGBoost	n_estimators	[100, 200, 300, 500]	200
		max_depth	[3, 5, 7, 9, 11]	7
		learning_rate	[0.01, 0.05, 0.1, 0.2]	0.2
		subsample	[0.6, 0.7, 0.8, 0.9, 1.0]	0.8
		colsample_bytree	[0.6, 0.7, 0.8, 0.9, 1.0]	0.8
		gamma	[0, 0.1, 0.2, 0.3]	0.2

		reg_alpha	[0, 0.1, 0.5, 1.0]	0
		reg_lambda	[0.5, 1.0, 1.5, 2.0]	1.0
		min_child_weight	[1, 3, 5, 7]	3
